# Arthroscopic Treatment Results of Triangular Fibrocartilage Complex Tears in Adolescents: A Systematic Review

**DOI:** 10.3390/jcm10112363

**Published:** 2021-05-27

**Authors:** Florian Schachinger, Sebastian Farr

**Affiliations:** Department of Pediatric Orthopaedics and Foot and Ankle Surgery, Orthopaedic Hospital Speising, Speisingertrasse 109, A-1130 Vienna, Austria; schachinger.florian@gmail.com

**Keywords:** wrist arthroscopy, TFCC, triangular fibrocartilage complex, hand

## Abstract

Introduction: Injury to the triangular fibrocartilage complex (TFCC) may cause chronic wrist pain and instability if left untreated. The current literature of adult cases suggests that arthroscopic treatment offers favorable outcomes and is associated with a low complication rate. This systematic review evaluated the outcomes of arthroscopic TFCC surgery in adolescents. Materials and Methods: A PRISMA-guided literature search of PubMed, Medline, Embase, Scopus, Cochrane Central Register of Controlled Trials, Cochrane Database of Systematic Reviews, and Cochrane Clinical Answers was conducted in May 2020. All studies reporting on (1) arthroscopic TFCC repair or debridement in (2) patients under the age of 19 years with (3) a minimum case number of four patients were extracted by two independent observers. The level of evidence of each study was assessed according to the Oxford Centre for Evidence-Based Medicine, and study quality was graded according to the Modified Coleman Methodology Score and the MINORS criteria. Clinical outcome scores, functional parameters, and any complications were reviewed. Results: The selected search terms initially resulted in a total of 986 possible articles. The authors eventually identified eight papers (all LoE IV) for inclusion in this systematic review. A total of 254 patients with verified TFCC tears and a mean age of 16 years (range, 7–19) received arthroscopic repair (162 patients, 67.1% of total) or debridement (77 patients, 29.7% of total). Arthroscopic treatment resulted in low pain levels, high patient satisfaction, and a fast return to sport. Complications overall were sparse and consisted mainly of persistent wrist pain (*n* = 31) and temporary paresthesia (*n* = 6) of the dorsal sensory branch of the ulnar nerve. Recurrent tears were sparse, with only four reported cases due to sports participation. Conclusion: Wrist arthroscopy is a reliable surgical option for treating TFCC tears in adolescents. The results obtained are comparable to those published in the literature. However, the variety of repair techniques and the low level of evidence across all included articles demand further prospective studies.

## 1. Introduction

In recent years, knowledge about the surgical treatment of TFCC tears has greatly increased. Various surgical techniques have been developed and their respective outcomes reported. Although arthroscopic treatment has become popular in the adult population, there is little information available on the treatment of TFCC tears in children and adolescents. As in adults, the main mechanism of injury is a fall onto a pronated and dorsally extended hand [1].

In 1986, Roth first described the basic technique of wrist arthroscopy and its application to treat ulnocarpal pain [2]. Soon thereafter, Osterman and Palmer reported their results on the arthroscopic treatment of TFCC tears [3,4]. Since then, various surgical options and repair techniques (e.g., all-inside, inside-out, and outside-in sutures) have been published with proof of the efficacy in adults, but there is still no consensus on a preferred technique [1,5,6,7,8,9,10,11,12,13,14]. Despite these technical advances, there is little evidence to be found on surgical treatment in children and adolescents. Only a few case series have described open [15] and arthroscopic surgery [16] to address TFCC tears in this immature population. These reports are limited by the uniformly retrospective nature of the studies, small sample sizes, and inconsistently reported outcome parameters.

The aim of this systematic review was therefore to evaluate the current evidence on wrist arthroscopic treatment of TFCC tears in exclusively young patients. We hypothesized that arthroscopic treatment is a viable option with high success and low complication rates in children and adolescents that present with arthroscopically verified TFCC tears.

## 2. Methods

### 2.1. Search Strategy

In May 2020, an online search was conducted in accordance to the PRISMA guidelines by the first and senior author of this study. PubMed, Medline, Embase, Scopus, Cochrane Central Register of Controlled Trials, Cochrane Database of Systematic Reviews, and Cochrane Clinical Answers were searched in duplicate for the terms “TFCC”, “triangular fibrocartilage complex”, “children”, “adolescents”, “treatment”, “repair”, “arthroscopy”, and “surgery”. In PubMed, the query was repeated with the inclusion of the appropriate MeSH terms. In Scopus, title, abstract, and keywords subheadings were included as well. The strategy was kept rather general to increase the potential number of results. All abstracts were checked separately by both authors, who were blinded to each other in their respective sessions. If an abstract met the inclusion criteria or insufficient information could be obtained, the full text was checked. Furthermore, the reference lists of all studies were checked for relevant articles. Thereafter, the authors compared their findings in a shared session to determine which articles would be chosen for final inclusion. The detailed search strategy is shown in the Supplementary Material.

### 2.2. Eligibility Criteria

The following criteria were pertinent for inclusion: original articles of all levels of evidence; articles presented in English or German; reports on patients aged 19 years or younger at the time of surgery; arthroscopic treatment (debridement or repair); and reporting clinical outcomes. The main outcome parameters searched for were pain levels (e.g., visual analogue scale (VAS)) and wrist range-of-motion (ROM) at the last available follow-up. Any reported outcome scores were considered as secondary outcome parameters in this study. Exclusion criteria were case reports or case series with less than 4 subjects, basic science studies (e.g., cadaver, biomechanical), studies reporting open surgery, data on adult patients (>19 years), missing or no detailed patient data for children, and adolescents in reports on mainly adult patients. 

### 2.3. Data Extraction and Study Quality Assessment

The following data were extracted and tabulated: demographics (e.g., age, sex, and side), tear type and classification [17,18], concomitant pathologies, surgical intervention (type of surgery, type of repair), pain level (VAS; whenever VAS values were not mentioned, terms such as “no pain”, “mild pain”, “moderate pain”, “serious pain” were considered), ROM, outcome scores (e.g., MMWS and DASH), and follow-up length. The methodological quality of the included studies was assessed according to a modified version of the Coleman Methodology Score [19] and the MINORS criteria [20]. Furthermore, the level of evidence according to the Oxford Center for Evidence-Based Medicine was obtained [21,22].

### 2.4. Statistical Analysis

Descriptive statistics are used to report the obtained results. Data of pediatric cases in adult reports were extracted for each presenting study. The results of the pooled data are reported in means/medians and ranges. We refrained from performing a comparative analysis and meta-analysis due to the heterogeneity of the patient-reported outcome scores used and the reported pre- and postoperative treatment algorithms.

## 3. Results

**Figure 1:**
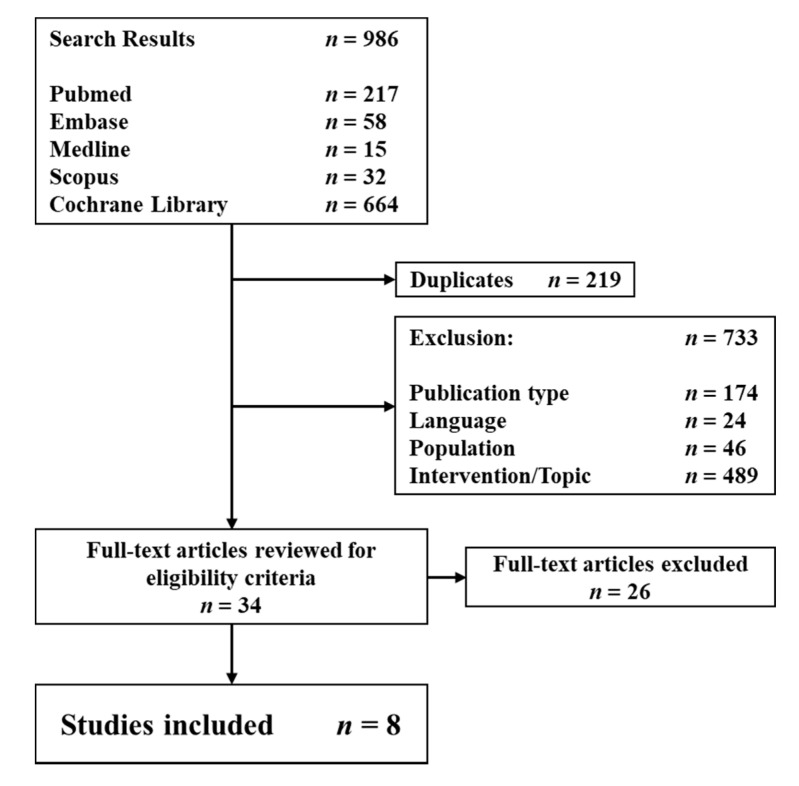
Flow chart of study inclusions and exclusions.

In total, 986 studies were obtained through the literature search (Figure 1). After the elimination of duplicates, wrong publication types, papers in languages other than English or German, and abstracts that did not meet the inclusion criteria, 34 full texts were checked for further eligibility in detail. Twenty-six papers were excluded after consensus due to incomplete patient data or presentation of exclusively open surgical approaches. Eventually, eight studies met the final inclusion criteria and were eligible for data extraction and analysis. All studies were Level IV studies, and none of them were controlled trials. All studies retrospectively reviewed patient charts. Three studies administered patient reported outcome scores over the telephone. If the patients were not contactable or refused to participate in the study, the last clinical follow-up was used [23,24,25]. Three studies had at least one prospective clinical follow-up [26,27,28]. The overall study quality was very low with MINORS criteria ranging from 10 to 13 and the Modified Coleman Score (MCS) ranging from 34 to 56. Six papers reported solely on pediatric and adolescent cases [23,24,25,27,28,29], whereas two studies also reported adult cases [26,30]. Terry and Waters [29] and Wu et al. [25] reported both open and arthroscopic cases, with data of the former being fully extractable. Five studies reported both arthroscopic repair and debridement. Two studies reported only cases with arthroscopic repair [27,30]. One study reported cases with only arthroscopic debridement [28]. Most authors used statistical analysis for both pre- and postoperative patient-reported outcome scores wherever available [24,25,26,27,28,29,30]. A comparison between the outcome of debrided and repaired cases was not conducted by any study.

### 3.1. Demographics

In total, the data of 254 patients (161 women, and 93 men) who received wrist arthroscopy of 259 wrists (133 left, 120 right wrists, and 6 no stated side) were reported. Patient age ranged from 7–19 years (Table 1). The vast majority (231 wrists, 89.2%) had traumatic TFCC tears according to their respective Palmer classification (Table 2) [17]. A clear history of a wrist trauma was reported for 163 patients (64.1%). Palmer’s TFCC tear classification was used in six studies [23,24,25,27,28,29]. The most common injury types reported were Palmer types 1B (*n* = 140, 54.1%) and 1D (*n* = 40, 15.4%). Multiple tears were present in 45 patients (range, 2–25). Shinohara et al. [30] used Atzei’s classification [18]. This system sub-divides Palmer type 1B tears. Thus, all extracted patients were categorized as such. McAdams et al. [26] described the tear according to its location being radial- or ulnar-sided. All extracted adolescent cases were ulnar-sided and therefore graded as Palmer type 1B tears in this systematic review. The time of the last follow up ranged from 2.4 to 168 months. All patients underwent clinical examination prior to surgery. All studies with the exception of Farr et al. (2015) [27] reported the use of MRI examination, with Fishman et al. [23] using MRI arthrography in 22/24 patients. X-ray examinations were obligatorily used by five studies [23,24,25,29,30]. Only Farr et al. (2018) [28] did not specifically report any preoperative examination algorithm. The majority of patients underwent conservative treatment before opting for surgery. Commonly, non-steroidal anti-inflammatory drug (NSAID) administration, splint/cast immobilization, physical therapy, and, in some cases, cortisone injections were applied. Terry and Waters and Wu et al. did not state specific conservative treatment methods prior to surgery [25,29]. Postoperatively, all patients were placed in a splint or cast for a period of up to 6 weeks. Terry et al. reported no specific postoperative treatment regimen [29]. McAdams and Fishman reported the results of patients who participated in high-level athletic sports [23,26]. Only McAdams exclusively reported isolated TFCC injuries in adolescents [26].

### 3.2. Debridement

Six studies reported the results of 77 patients who received TFCC debridement as a primary intervention. Fishman et al. [23] did not specifically declare the distribution of cases who received debridement or repair, but reported debridement of central- and radial-sided tears. Farr et al. (2018) [28] reported the largest single cohort of 13 patients who solely received TFCC debridement. They primarily debrided 1A (*n* = 3) and 2C (*n* = 2) tears, but also occasionally stable 1B (*n* = 2) and 1D (*n* = 1) tears with punches and shaver devices. They stated that the latter two were cases that would receive TFCC repair nowadays. In a few cases with ulnar positive variance, an ulnar shortening osteotomy (USO) was performed concomitantly. Wu et al. [25] primarily debrided 1A tears (14 wrists) as well as partial 1C (*n* = 1) and 1D (*n* = 11) tears. The total number of combined tear types that received sole debridement was not specifically stated. Trehan et al. [24] debrided 13 cases. Terry and Waters [29] reported two cases (1A, 1D) and McAdams et al. [26] reported one case (1B) who received debridement.

### 3.3. Repair

Seven studies reported the results of a combined number of 162 patients who received arthroscopic repair [23,24,25,26,27,29,30]. Fishman et al. [23] did not specifically state how many wrists were arthroscopically repaired, but stated that all ulnar-sided injuries underwent repair. Thus, patients with Palmer Type 1B and 1A/B tears were counted as arthroscopically repaired. Equally, Wu et al. [25] reported 105 cases who received arthroscopic repair and 21 that were treated with an open approach. Nine patients received both debridement and repair. Several techniques were reported. Six studies used variations in outside-in techniques [23,24,25,28,29,30]. Shinohara et al. [30] reported the repair of foveal tears using a transosseous suture technique. McAdams et al. [26] were the only ones who used an inside-out technique. Farr et al. [27] (*n* = 12) and Shinohara et al. [30] (*n* = 4) were the only studies that exclusively reported the outcome of arthroscopic repairs. All of them were Palmer type 1B tears.

### 3.4. Concomitant and Subsequent Surgeries

Overall, 21.6% (*n* = 56) of all wrists received USOs due to static or dynamic ulnar positive variance. In 16.2% (*n* = 42) of cases, an ulnar styloid non-union excision was performed, and 5.8% (*n* = 15) had DRUJ stabilization surgery. In 15.8% (*n* = 41) of cases, some other form of concomitant surgery (e.g., SL-ligament debridement, thermal shrinking of intercarpal ligaments, etc.) was performed. No concomitant procedure was performed in 33.6% (*n* = 87) of all wrists. These cases may be counted as isolated TFCC tears. However, only four studies specifically indicated that their cases had isolated TFCC tears without any concomitant condition or injury (*n* = 33) [23,26,29,30]. Wu et al. [25] performed USOs in patients with an ulnar positive variance of 1 mm or greater. Fishman et al. [23] performed USOs prior to wrist arthroscopy and tried to achieve a neutral to slightly negative ulnar variance to a maximum of −2 mm.

### 3.5. Complications

Complications varied among all studies. The majority of patients with complications suffered from ongoing or unresolved wrist pain (*n* = 32) and temporary paresthesia (*n* = 4) of the dorsal sensory branch of the ulnar nerve. Three studies reported no postoperative complications or other adverse events [24,26,29]. Terry and Waters [29] had one patient with persistent pain due to Sudeck’s disease, which was already present before surgery and was therefore not counted as a complication. Fishman et al. [23] reported four cases with delayed wound healing which resolved on their own without any further intervention.

### 3.6. Outcomes

All studies reported clinical data and outcome scores (Table 3). Five studies reported ROM percentages of the affected wrists. Three studies reported the ROM based on the MMWS score with all patients achieving excellent results [23,29,30]. Farr et al. (2015) [27] and Farr et al. (2018) [28] reported the postoperative ROM in relation to the uninjured contralateral side (range 87–98 flex/ext, 82–93% add/abd, and 89–100 pro/sup). Five studies [25,27,28,29,30] used the Modified Mayo Wrist Score (MMWS) with postoperative values ranging from a mean of 88 to 97.5. Wu et al. [25] reported median and IQR values. Postoperative MMWS values were 100 (IQR 95–100) for TFCC treatment with concomitant bony procedures and 95 (IQR 85–100) for patients who only had soft tissue procedures. Four studies [24,26,27,28] used the Disability of the Hand and Shoulder (DASH) score with mean values from 0 to 17. Only Farr et al. (2015) [27] and Farr et al. (2018) [28] used the VAS for pain assessment at the last clinical follow-up. The VAS values improved from a mean of 7.0 (range 2–10) to 1.7 (range 0–5) in patients who received Palmer type 1B repair and from 5.7 (range 3–9) to 1.8 (range 0–6) in patients who underwent TFCC tear debridement. Two studies used verbal terms for pain assessment, but all of them were in accordance with the terminology of the MMWS. Shinohara et al. [30] used the Hand20 outcome questionnaire; however, out of five adolescent patients, the score of only one patient was available. Fishman et al. [23] opted for a questionnaire based study using the Pediatric Outcomes Data Collection Instrument (PODCI). Twenty out of twenty-two patients participated in the telephone survey. The individual mean scores were 97 (range 75–100) for upper extremity function, 91 (range 54–100) for sports and physical functioning, 73 (range 22–100) for pain and comfort, and 89 (range 60–100) for overall happiness.

## 4. Discussion

The current evidence on open versus arthroscopic treatment of TFCC tears is somewhat inconclusive, with satisfying surgical results for both approaches [31,32]. Clinical examination, X-rays, and MRI examination may be helpful in opting for the use of wrist arthroscopy. However, wrist arthroscopy is considered the diagnostic gold standard; therefore, it is obvious that arthroscopic repair should be attempted in the same operation [33,34,35].

All studies reported satisfying outcomes for arthroscopic debridement and repair of TFCC tears. In general, the indication for each treatment is dependent on the localization as well as the etiology (traumatic, chronic wear) of the tear. Lesions of the central portion may be treated by sole debridement, as the stabilizing function on the DRUJ is not compromised [36,37]. In cases with complete ulnar-/radial-sided or even foveal tears, repair should be achieved to ensure DRUJ stability [38].

A comparison between arthroscopic debridement and repair was not deemed reasonable as most studies reported both interventions without distinguishing the two cohorts. Additionally, the number of cases was too low and the type of treatment is dependent on the location of the tear. Interestingly, the studies published by Farr et al. (2015) [27] and Farr et al. (2018) [28] were suitable for a descriptive comparison as both studies included cases performed by the same surgeon. Patients with arthroscopic debridement had lower mean pre- and postoperative MMWS values (65 vs. 70, 88 vs. 90), better mean postoperative ROM and better mean postoperative grip strength (86% vs. 87%). This may be due to a lower injury severity in cases who received sole debridement, as both cohorts received concomitant procedures (e.g., USO, thermal ligament shrinking). Furthermore, recent data suggest that the role of a positive ulnar variance on the outcome of arthroscopic TFCC treatment could be overestimated [39,40].

Three studies reported isolated pre- and postoperative pain levels [27,28,29] and five studies reported isolated postoperative pain levels [26,27,28,29,30]. The vast majority of patients had no pain and a few had moderate pain levels. Unfortunately, only Farr et al. [27,28] used VAS levels. Three studies used the pain levels from the MMWS [26,29,30]. All other studies did not specifically state isolated pain levels. Terry and Waters [29] reported one patient who had severe persisting pain. However, this particular patient already suffered from a complex regional pain syndrome type 1 preoperatively. Overall, the reported pain levels were low and patient satisfaction was high. The reported outcome methods varied widely among all studies. Most used the MMWS [25,27,28,29,30], two used the DASH [27,28], two used the Quick-DASH [24,26], and one used the PRWE [25]. Shinohara et al. [30] primarily used the Hand20 score, but only one of all pediatric patients completed the score.

All but two studies [26,30] reported the outcomes of patients who received subsequent or concomitant surgeries apart from TFCC repair or debridement. According to Wu et al. [25], patients with sole ligamentous injuries had poorer outcomes than patients with additional bony procedures. A high percentage received concomitant or subsequent surgical procedures. USOs were performed in 21.6% (*n* = 56) of all procedures. The second most common procedure was the excision of ulnar styloid non-unions. However, the vast majority of these patients come from the cohort reported by Wu et al. who accounted for 58.7% (*n* = 149) of all patients who received surgery. Overall, it is still common consensus that many patients need concomitant or subsequent procedures because of various other pathologies. Patients with a positive ulnar variance are especially prone to sustaining TFCC injuries and may benefit from an USO [41,42].

A detailed and strict conservative treatment algorithm prior to surgical intervention was only reported by Trehan et al. [24]. The vast majority used various conservative treatment methods. However, all but Terry and Waters [29] reported some form of conservative treatment regimen before opting for surgery. Wu et al. [25] reported a previous fracture of the radius and/or ulna in 55.6% (*n* = 85), ulnar styloid non-unions in 32.6% (*n* = 50) and DRUJ instability in 13.7% (*n* = 21) of all cases. Only 24.8% (*n* = 38) of all wrists underwent isolated TFCC treatment. Thus, there is a clear indication that TFCC tears are often associated with additional injuries, but their reported results do not necessarily solely display the outcome of the TFCC treatment, but rather the outcome of all procedures combined.

In comparison with the current literature on adult cases, the presented results in children and adolescents may be considered similar overall [43,44,45]. However, as in the analyzed adolescent cohort, many different techniques and approaches have been reported in adults so far. The current consensus is to debride Palmer type 1A tears and repair type 1B tears to either the capsule or fovea in unstable cases [13,18,46]. Less common tears, types 1C and 1D, can be addressed by sole debridement or transradial repair, respectively [47,48]. Although these recommendations currently do not differ between children and adults, young athletes in competitive sports may have different demands and outcome expectations than the common population. Prospective randomized controlled trials with a strict preoperative treatment algorithm may help define patient-oriented treatment paths for patients of all levels of activity.

The study is mainly limited by the low study quality of the included reports. There were no uniform outcome scores or outcome measurement parameters applied, which is attributed to the lack of validated outcome measures for children under the age of 16 years. Moreover, observer bias was eminent such as in the Wu et al. [25] and Fishman et al. [23] reports, which gathered their last follow-up outcome data primarily via telephone or retrospective clinical chart review. Although detailed instructions were provided, patients cannot be considered healthcare professionals and therefore ROM and pain assessments might be inaccurate. We acknowledge a certain risk of bias, as all of the included studies were uncontrolled case series with a broad spectrum of concomitant injuries. However, the majority of patients had undergone multiple conservative treatment methods that eventually failed. Independent of the underlying pathology, we think that conservative treatment is the primary step in the TFCC treatment algorithm. Moreover, more than half of all the included patients were from the cohort reported by Wu et al. [25]. We do not think there is a significant risk of bias, as the other included studies reported comparable outcomes and no formal meta-analysis was conducted. In conclusion, the reported outcomes for arthroscopic treatment of TFCC tears in adolescents were uniformly good to excellent. Complications occurred in only a few cases, and overall patient/parent satisfaction was high.

## Figures and Tables

**Table 1:** Key aspects.

Author	Study Design (LoE)	Patient Count (Female)	Side	Mean Age at Surgery (Years) (Range)	Debridement	Repair	Mean Follow up (Years)	Modified Coleman	MINORS
Terry and Waters [29]	Retrospective case series (IV)	6 (4)	n.s.	15.8 (14–17)	2	4	1.5	34	12
McAdams et al. [26]	Retrospective case series (IV)	5 (3)	2L/3R	18.0 (16–19)	1	4	2.1	57	12
Shinohara et al. [30]	Retrospective case series (IV)	4 (0)	2L/2R	16.5 (15–18)	0	4	2.9	51	12
Farr et al., 2015 [27]	Retrospective case series (IV)	12 (8)	5L/7R	16.3 (13–19)	0	12	1.3	59	13
Farr et al., 2018 [28]	Retrospective case series (IV)	13 (12)	8L/5R	15.6 (11–18)	13	0	6.7	65	13
Fishman et al. [23]	Retrospective case series (IV)	22 (16)	11L/11R	14.2 * (11–17)	n.s.	n.s.	1.6	49	10
Trehan et al. [24]	Retrospective case series (IV)	43 (32)	22L/22R	15 (10–17)	13	31	7	54	12
Wu et al. [25]	Retrospective case series (IV)	149 (86)	83L/70R	15.5 ** (7–19)	40	109	1.8 **	44	13

* = age at presentation, ** = median, n.s. = not specifically stated.

**Table 2:** Palmer classification.

Author	1A	1B	1C	1D	Combined
Terry and Waters [29]	1	4	0	1	0
McAdams et al. [26]	0	5 *	0	0	0
Shinohara et al. [30]	0	4 *	0	0	0
Farr et al. 2015 [27]	0	12	0	0	0
Farr et al. 2018 [28] **	3	2	0	1	5
Fishman et al. [23]	3	10	0	5	4
Trehan et al. [24]	7	23	0	3	11
Wu et al. [25]	15	79	1	30	25

* = classified based on anatomical tear description ** = 2 cases with Palmer Type 2 tears not included in this table.

**Table 3:** Clinical outcomes.

Author	Repair Technique	Additional Procedures (*n*)	Post-OP Immobilization	Pain	Full ROM (%)	Complications (%)	Post-OP MMWS	Post-OP DASH
Terry and Waters [29]	Outside-in	3	none stated	5 none 1 moderate *	100	0	97.5 (85–100)	n.a.
McAdams et al. [26]	Inside-out	0	6 weeks (2 weeks sugar-tong, 4 weeks short-arm)	VAS 0 (0–0)	n.a.	0	n.a.	0 *** (0–0)
Shinohara et al. [30]	Outside-in, transosseus	0	4 weeks long-arm	3 none, 1 mild *	100	25 (recurrent pain)	96.25 (90–100)	n.a.
Farr et al., 2015 [27]	Outside-in	10	6 weeks (2 weeks long-arm, 4 weeks short-arm)	VAS 1.7 (0–5)	87 (flex/ext to contralateral side)	25 (paresthesia)	88 (75–100)	16 (0–40)
Farr et al., 2018 [28]	Debridement	6	2 weeks padded dressing	VAS 1.8 (0–6)	93 (flex/ext to contralateral side)	15 (recurrent pain)	90 (80–100)	17 (0–47)
Fishman et al. [23]	Outside-in	7 (USOs, rest not stated)	4 weeks long-arm cast (debridement), 6 weeks long-arm cast (repair)	73 (22–100; PODCI subsection)	100	25	n.a.	n.a.
Trehan et al. [24]	Outside-in	9	none stated	n.a.	n.a.	2 (recurrent pain)	n.a.	4 *** (0–21)
Wu et al. [25]	Outside-in	118	6 weeks (4 weeks long-arm, 2 weeks short-arm)	23 moderate/severe *	85	19 (recurrent pain)	95 ** (IQR 85–100)	n.a.

* = MMWS terminology used, ** = median, *** = Quick-DASH.

## Data Availability

No new data were created or analyzed in this study. Data sharing is not applicable to this article.

## Supplementary Materials

The supplementary material are available online at https://www.mdpi.com/article/10.3390/jcm10112363/s1.
