# Supplementary material for: Arthroscopic Treatment Results of Triangular Fibrocartilage Complex Tears in Adolescents: A Systematic Review
Source: J Clin Med. 2021 May 27;10(11):2363. doi: 10.3390/jcm10112363 (PMC8199377; doi:10.3390/jcm10112363)
Supplement: Supplementary file 1 [file jcm-10-02363-s001.zip › jcm-1173034-supplementary.pdf]

**Pubmed:**

(TFCC OR triangular fibrocartilage complex) AND (children OR adolescents) AND (arthroscopy OR surgery OR treatment)
217 hits

(TFCC OR triangular fibrocartilage complex[MeSH Terms]) AND (children OR adolescents) AND (arthroscopy OR surgery OR treatment)
143 hits

(TFCC[MeSH Terms] OR triangular fibrocartilage complex) AND (children OR adolescents) AND (arthroscopy OR surgery OR treatment)
The following term was not found in PubMed: TFCC[MeSH Terms], 200 hits

(TFCC OR triangular fibrocartilage complex) AND (children OR adolescents) AND (arthroscopy[MeSH Terms] OR surgery OR treatment)
213 hits

(TFCC OR triangular fibrocartilage complex) AND (children OR adolescents) AND (arthroscopy OR surgery[MeSH Terms] OR treatment)
198 hits

(TFCC OR triangular fibrocartilage complex) AND (children OR adolescents) AND (arthroscopy OR surgery[MeSH Terms] OR treatment[MeSH Terms])
160 hits

**Embase:**

(tfcc OR 'triangular fibrocartilage complex'/exp OR 'triangular fibrocartilage complex' OR (triangular AND ('fibrocartilage'/exp OR fibrocartilage) AND ('complex'/exp OR complex))) AND ('children'/exp OR children OR 'adolescents'/exp OR adolescents) AND ('arthroscopy'/exp OR arthroscopy OR 'surgery'/exp OR surgery OR 'treatment'/exp OR treatment)
58 hits

**Medline:**

(TFCC OR triangular fibrocartilage complex) AND (children OR adolescents) AND (arthroscopy OR surgery OR treatment).mp
15 hits

**Cochrane Library (RCT, Database of Systematic Reviews, Clinical Answers):**

(TFCC or triangular fibrocartilage complex).mp. [mp=ti, ot, ab, tx, kw, ct, sh, hw]
32 hits

**Scopus:**

TITLE-ABS-KEY ( ( tfcc OR triangular AND fibrocartilage AND complex ) AND ( children OR adolescents ) AND ( arthroscopy OR surgery OR treatment ) )
146 hits

ALL ( ( tfcc OR triangular AND fibrocartilage AND complex ) AND ( children OR adolescents ) AND ( arthroscopy OR surgery OR treatment ) )
664 hits

(ALL ( tfcc OR triangular AND fibrocartilage AND complex ) AND ALL ( children OR adolescents ) TITLE-ABS-KEY ( arthroscopy OR surgery OR treatment ) )
460 hits
